# Evaluation of different b-values in DWI and ^1^H MRS for pancreatic cancer and pancreatitis: a rabbit model

**DOI:** 10.1042/BSR20181933

**Published:** 2019-11-15

**Authors:** Liguo Hao, Lijie Liu, Xin Meng, Guanghao Yu, Enbang Li, Hongqian Gu

**Affiliations:** 1 Medical Technology School of Qiqihar Medical University, Qiqihar, Heilongjiang, People’s Republic of China; 2 Centre for Medical Radiation Physics, University of Wollongong, NSW, Australia; 3 College of Life Science and Agriculture Forestry, Qiqihar University, Qiqihar, Heilongjiang, People’s Republic of China; 4 Third Affiliated Hospital of Qiqihar Medical University, Qiqihar, Heilongjiang, People’s Republic of China; 5 Medical Imaging School of Mudanjiang Medical University, Mudanjiang, Heilongjiang, People’s Republic of China

**Keywords:** b value, diffusion weighted imaging, mass pancreatitis, Pancreatic cancer, rabbit model

## Abstract

Pancreatic cancer is a common malignant tumor with high incidence of metastasis. Currently, there is no absolute standard for the choice of b-value for diffusion-weighted imaging (DWI) for pancreatic cancer. The b-value is rarely reported in animal model study, especially in pancreatic cancer/mass pancreatitis rabbit models. The authors’ aim was to determine the different b-values to differentiate the diagnosis of pancreatic cancer and mass pancreatitis in rabbit models using DWI. When comparing the effect of different b-values in diagnostic process, the pathological results could be regarded as the gold standard. In this research, 30 healthy New Zealand rabbits were selected and divided into three groups by random number table method: group 1 (pancreatic cancer), group 2 (mass pancreatitis) and the control group (healthy). After DWI (three different b-values 333, 667, 1000 s/mm^2^, respectively) and MRI examination, the model rabbits were then killed. Afterward, the tumor mass was removed for biopsy, and occupation anatomy and tumor histopathology were examined. Fat-suppressing sequences of T_2_WI, DWI, ADC, difference of ADC (DADC), and MRS were used. The present study determined that the effective differential diagnosis of pancreatic cancer and pancreatitis was determined at low b-values (333 s/mm^2^) when performed DWI inspection in rabbit models.

## Introduction

Pancreatic cancer is a common tumor and a highly malignant cancer in human. Pancreatitis is a common non-malignant disease that affects the same organ, pancreas. However, each disease has its own pathophysiological and molecular biological development process. An accurate measure of pancreatic cancer incidence is still unclear, due to the difficulty of timely diagnosis, it is reported that 40–45% of the patients with pancreatic cancer have no chance for surgery at initial diagnosis [1]. Conventional MRI can accurately diagnose pancreatic cancer and evaluate the resectability of pancreatic cancer before surgery. The diffusion-weighted imaging (DWI) sequence can reflect the characteristics of microstructure changes, and is frequently used as the early detection approach for pancreatic cancer.

The diffusion-sensitive gradient field parameter applied in DWI is expressed as b-value [2]. The apparent diffusion coefficient (ADC) value reflects changes in tissue cells, and is negatively correlated with changes in cells within the tissue. DWI differs between organizations mainly by a different b-value. Theoretically, the higher b-value, the closer the signal appears to the real tissue ADC value. Although 3.0 T MRI has good imaging properties for our purposes, visceral artifacts of the upper abdomen appear on the DWI sequence, which affects the measurement of ADC value [3]. This drawback leads to errors in the region of interest (ROI); within the blood vessels, pancreatic duct, and the spatial resolution, signal to noise ratio and other effects, the final ADC value therefore does not accurately reflect the tumor’s pathological conditions. DADC (difference of ADC) was therefore selected to observe the different tissues. Most publications concerning b-values in the past focused on human study. In animal models, mice were the most commonly examined subject [4]. Nevertheless, rabbit pancreatic models were rarely reported. In the current study, pancreatic cancer and chronic pancreatitis models were compared with normal rabbits as the control group. ADC and DADC values were observed in the diagnosis of each rabbit model by different b-values in DWI. Changes in ^1^H MRS in the rabbit models were also evaluated.

The reason for choosing rabbit model for study is stated below. At first, the model is more intuitive and effective than mice model. Second, the quality of image is better than mice. At last, imaging of rabbit model is closer to human body. The advantage of the present study is first discussed on the application of ADC values and DADC values in the model of pancreatic tumor under different b-values; and second, establishing a reference method for researchers to conduct similar experiments in the future.

## Materials and methods

### Animal model preparation

In the present study, 30 healthy New Zealand rabbits were selected, 15 males and 15 females, aged 4–5 months, weighing 2–3 kg each. They were subcage raised and provided by the Animal Experimental Center of Qiqihar Medical University (China). The ambient temperature was 15–25°C and relative humidity was constant between 40 and 70%. Drinking water and special standard feed were provided to the experimental animals for consumption after sterilization. The present study was approved by the Ethics Committee of Qiqihar Medical University of China, which agreed to choose the experimental animals for research (approval number: 201403041).

Preparation reagents and instrument for the animals included hair removal agent (sodium sulfide and distilled water 1:9), 75% alcohol, uralan (urethane, serial number: 20120327, Sinopharm Group Chemical Reagent), anion iodine solution, 0.9% physiological saline, injection of aztreonam (serial number: 20150321, Hainan Zhonghua Pharmaceutical Industry), injection of Astragalus polysaccharide (serial number: 150301, Kangerhao of Sichuan Pharmaceutical), and injection of gemcitabine (serial number: 150512, Jiangsu Haosen Pharmaceutical). Animal manipulation was performed by bending, straight cutting, tweezers, surgical pliers, surgical knife handle, surgical blade, surgical pull hook, needle holder, surgical needle, thread, aseptic console, cotton, and gauze.

Experimental animals which were established model were carried out in molecular imaging laboratory of Qiqihar Medical University (China). The experimental rabbits were divided into three groups by random number table method: group 1 (pancreatic cancer), group 2 (mass pancreatitis) and the control group (healthy). Each group had five males and five females, with no significant differences in weight and age of rabbits.

The rabbits in group 1 were implanted with VX-2 pancreatic cancer tumor tissue (from the Molecular Imaging Research Center of the Fourth Affiliated Hospital of Harbin Medical University, China). Two original tumors implanted in the bilateral thigh muscles of rabbits were prepared with VX-2 [5,6]. The rabbits were killed when the tumor grew to 3–5 cm as measured by ultrasound. The tumor tissue was taken under aseptic conditions and placed in sterile Petri dishes, and rinsed repeatedly with 0.9% physiological saline. Necrotic tissues and surrounding blood vessels were removed. Gray-white fish-like tissues blocks were cut out into pieces of approximately 0.5–1 mm^3^ in volume with a scalpel. Tumor dilution was made by adding physiological saline into the mix. The tumor tissue mass dilution was extracted by using a 50-ml syringe needle connected to a 2.5-ml syringe [7].

Before sample preparation, each animal was forbidden from drinking water 8 h prior, and fasted for 24 h (for ten healthy rabbits). Urine dilution was performed by ear vein injection with 20% urethane (urethane dose: weight (kg) × 5 ml/kg). The rabbits were anesthetized and fixed on the experimental bench, with skin disinfected by iodine solution. A 3–4 cm cut was made along the vertical white line 2 cm under the xiphoid. Once the pancreas was located along the duodenum, it was punctured with a scalpel 2 cm from the duodenum nipple. The exposed pancreas was then injected with the tumor dilution by using 1-ml syringes. Finally, the capsule of pancreas was sutured.

The ten rabbits in group 2 were set as the chronic pancreatitis model. They were forbidden from drinking water 8 h before the procedure, and fasted for 24 h prior to operation. The laparotomy method used was the same as group 1. We also used the common pancreatic duct ligation method, with the rabbits’ main pancreatic duct incompletely ligatured in the pancreas neck [8,9], together with the main pancreatic duct and 3-0 polypropylene suture (double-stranded by 3-0 silk). The 3-0 polypropylene suture was then removed, so that more than 75% of the main pancreatic duct was ligatured. Thus, the chronic mass pancreatitis was formed.

The successfully modeled rabbits were fed in the Animal Center of Qiqihar Medical University. Anti-inflammatory treatment was performed by using mixed aztreonam (4.0 g aztreonam and 0.9% physiological saline 100 ml), 30 ml/day/each rabbit. Three weeks later, they were finally examined by MRI.

The ten rabbits in the control group were given no treatment.

In order to observe the tumor growth, the rabbits after modeling were monitored by ultrasound. MRI examination was performed after 3 weeks, and the model rabbits were killed immediately. The mass was then removed for biopsy.

### Detection methods

The MRI machine used was a GE Discovery MRI 750 3.0 Tesla Superconducting Magnetic Resonance Imaging in the Department of MR at the Third Affiliated Hospital of Qiqihar Medical University. This system contains an 8-channel phased array coil, respiratory triggering, with a magnetic field strength of 3.0 T, and FOV: 395–430 mm. Three different b-values were selected (333, 667, 1000 s/mm^2^), taken in two different directions simultaneously in the diffusion direction.

MRS has an advantage in avoiding the adipose tissue surrounding the pancreas when selecting the volume of interest (VOI) in the pancreatic cancer model. The voxel size 8 × 8 × 8 mm^3^ was adjusted appropriately according to the pancreas size. Image acquisition time was 150 s.

In order to eliminate the effects of fat around the pancreas, a saturation band was set around the VOI. Water was inhibited by performing regular automatic pre-scanning before magnetic resonance spectroscopy. Spectral imaging was performed with line width (LW) < 25 Hz, and water inhibition rate > 80%. At least one satisfactory VOI spectral dataset was obtained in each group of pancreatic cancer model.

### Data collection

All the data were processed with GE Advantage Workstation using Volumeshare 5 software. The ADC values were measured in normal pancreatic parenchyma. The total solid area of pancreatic cancer lesions, and chronic mass pancreatitis were measured under DWI sequence imaging with different b-values (333, 667, 1000 s/mm^2^). ADC values were measured on fitted ADC images using selected circles to define each ROI. It was necessary to make the ROI as large as possible to include the great extent of target organ. It was necessary to avoid artifacts (near the edge of the lesion, cystic necrosis areas, and in the pancreas tissue area). The ADC values were measured at three different levels separately in two groups. Each measurement area of interest was kept at the same size (as much as possible) and the mean ADC value taken (after the average ADC value was calculated with three values). Areas with liquefaction necrosis in the middle of the pancreas lesions were avoided. Note that ROI selection should be chosen surrounding the parenchyma of the pancreas. The ROIs measured were 20, 46, and 100 mm^2^. The obtained MRS data were post-processed by SAGE 7.0 image post-processing software.

### Data analysis

SPSS (v17.0, SPSS Inc., Chicago, IL, U.S.A.) was used for statistical analysis in the present study. Specifically, Shapiro–Wilk’s test was used to assess normality assumptions. Interclass correlation coefficient (ICC) and Bland–Altman analysis were used to evaluate the tumor ADC measurements by the same reader. The coefficient of variance (CV) was used to quantify the measurement errors, using 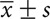 to express the normal distribution of measurement data. Student’s *t* test was used to compare measurement information between groups. We used χ^2^ test to compare the counting data, *P*<0.05 was considered to be a statistically significant result. Bonferroni correction was used to correct for the accumulation of the α error when more than two groups were compared.

## Results

### General situation

After 3 weeks, MRI examination was performed. At this time point, two rabbits were dead after modeling in group 1, i.e. the death rate of pancreatic cancer model rabbits was 20%. Meanwhile in group 2, ten chronic pancreatitis model rabbits survived, i.e. the death rate of chronic mass pancreatitis model rabbits was zero. Ten rabbits in the control group were growing healthily. After examination with MRI, eight image datasets of pancreatic cancer model rabbits in group 1, ten image datasets of pancreatitis model rabbits in group 2, and ten image datasets of normal pancreatic rabbits in the control group were successfully obtained.

### Differences of MRI in pancreatic cancer and pancreatitis

**Figure 1:**
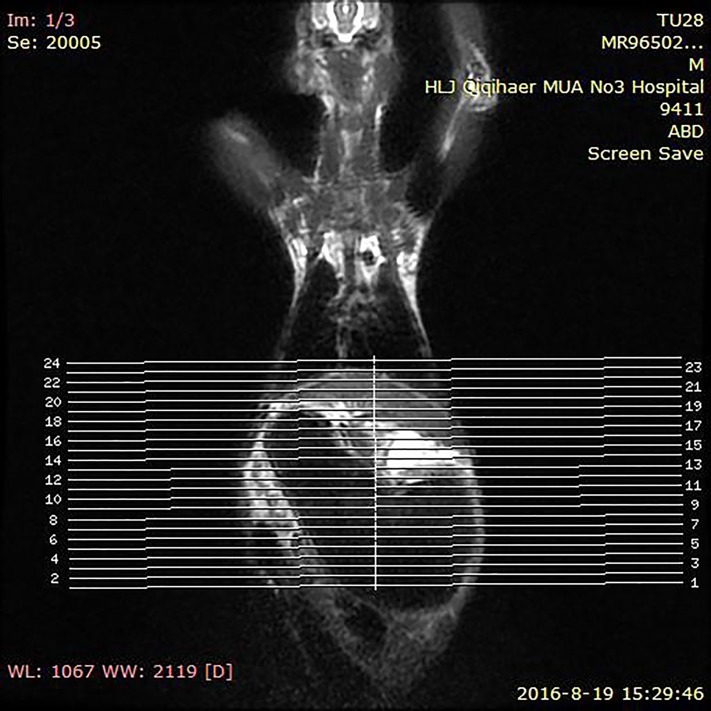
MRI scan positioning phase of model rabbit Determine the scanning positioning of the Magnetic Resonance Image.

Since the pancreas of model rabbit is located behind the stomach, the upper bound scanning positioning phase selection range was diaphragmatic. The lower bound was set as the iliac spine connection (Figure 1).

**Figure 2:**
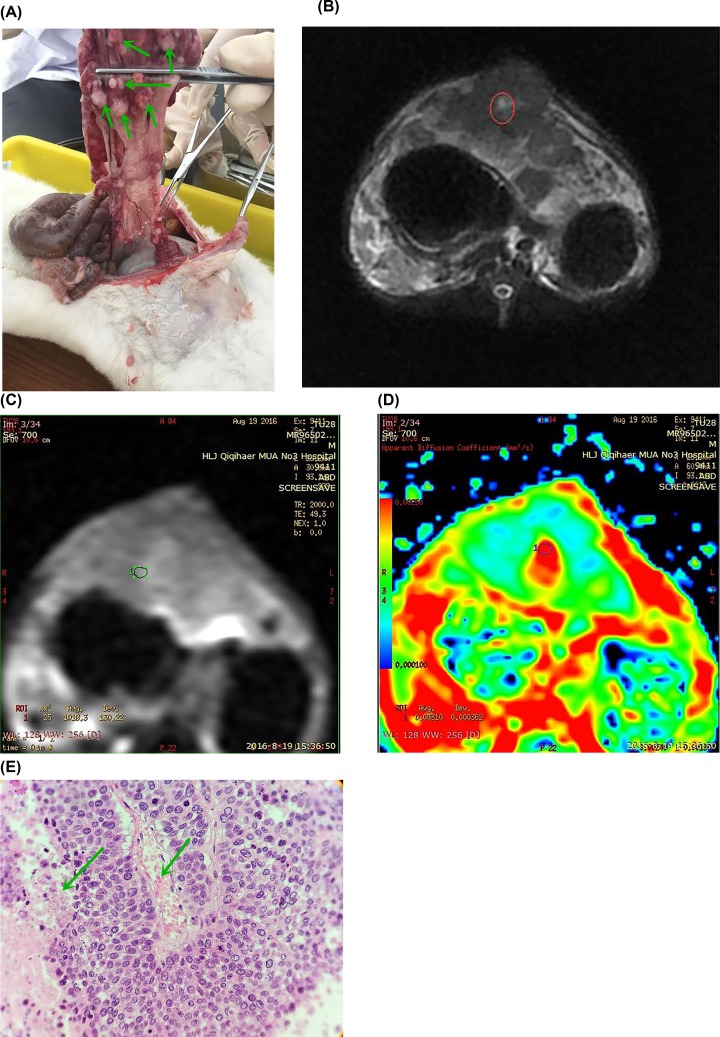
The data of pancreatic cancer model rabbit in group 1 (**A**) There were multiple tumors around pancreas tissues. (**B**) T_2_WI image, slightly longer T_2_ signal in the lump-like tissues, pancreatic cancer showed more clearer boundaries, the signal was relatively uniform. (**C**) DWI image, the lesion showed an uneven, slightly higher signal. (**D**) b = 333 s/mm^2^, ADC = 3.10. (**E**) The left arrow indicates tumor necrosis. The right arrow indicates angiogenesis, constituting a false nipple-like growth.

For the pancreatic cancer model rabbits (group 1), there were multiple tumors around pancreas tissues in the pancreas area (Figure 2A). During imaging, a fat-suppressing sequence of T_2_WI (Figure 2B) was used. Slightly longer T_2_ signal in the lump-like tissues, and high signal lesion in the central necrotic area were observed. Pancreatic cancer showed more clearer boundaries in MRI images, and the signal was relatively uniform. In the DWI image (Figure 2C), the lesion showed an uneven, slightly higher signal. The b-value was 333 s/mm^2^ and ADC value was 3.10 in the lesion area (Figure 2D). In the pathological image shown in Figure 2E, HE staining can be seen at a level of 400 times. The left arrow indicates tumor necrosis. The right arrow indicates angiogenesis, constituting a false nipple-like growth.

**Figure 3:**
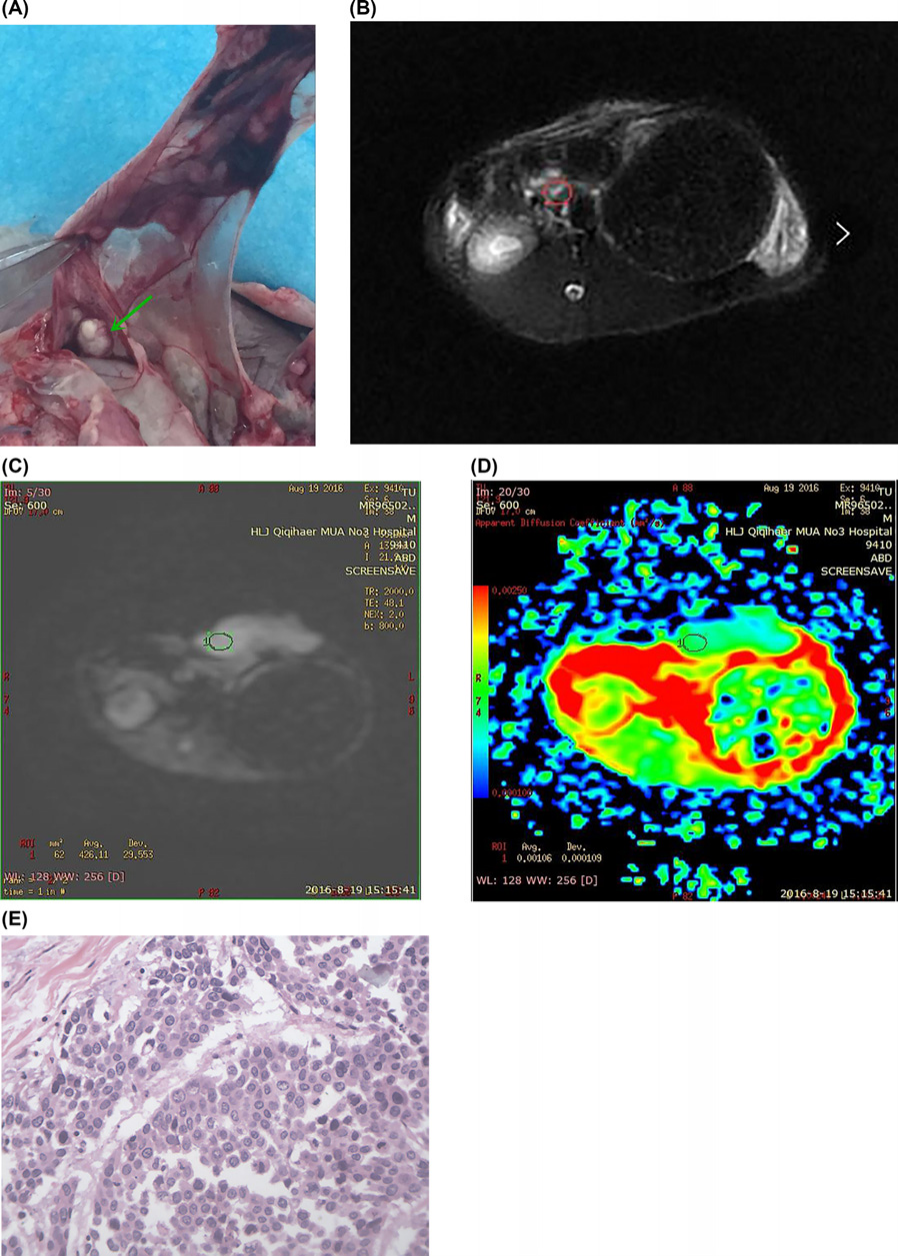
The data of pancreatitis model rabbit in group 2 (**A**) A round-like placeholder. (**B**) T_2_WI image, the pancreas border was blurred, slightly longer T_1_ and T_2_ signal, uneven signal. (**C**) DWI image, an uneven slightly higher signal. (**D**) b = 333 s/mm^2^, ADC = 1.06. (**E**) Chronic inflammatory cells infiltrated and interstitial fibrous tissue hyperplasia.

Pancreatitis model rabbit (group 2) images are shown in Figure 3. In the model rabbit anatomy image (Figure 3A), a round-like placeholder was found in the pancreatic area. For the fat-suppressing sequence of T_2_WI (Figure 3B), the pancreas border was blurred, with a slightly longer T_1_ and T_2_ signal, yielding an uneven signal. The DWI image (Figure 3C) showed an uneven slightly higher signal in the lesion area signal. The b-value was 333 s/mm^2^, and ADC value in the lesion area was 1.06 (Figure 3D). The pathological image shown in Figure 3E had HE staining, 400×, with necrosis in local tissue, chronic inflammatory cells infiltrated and interstitial fibrous tissue hyperplasia.

**Figure 4:**
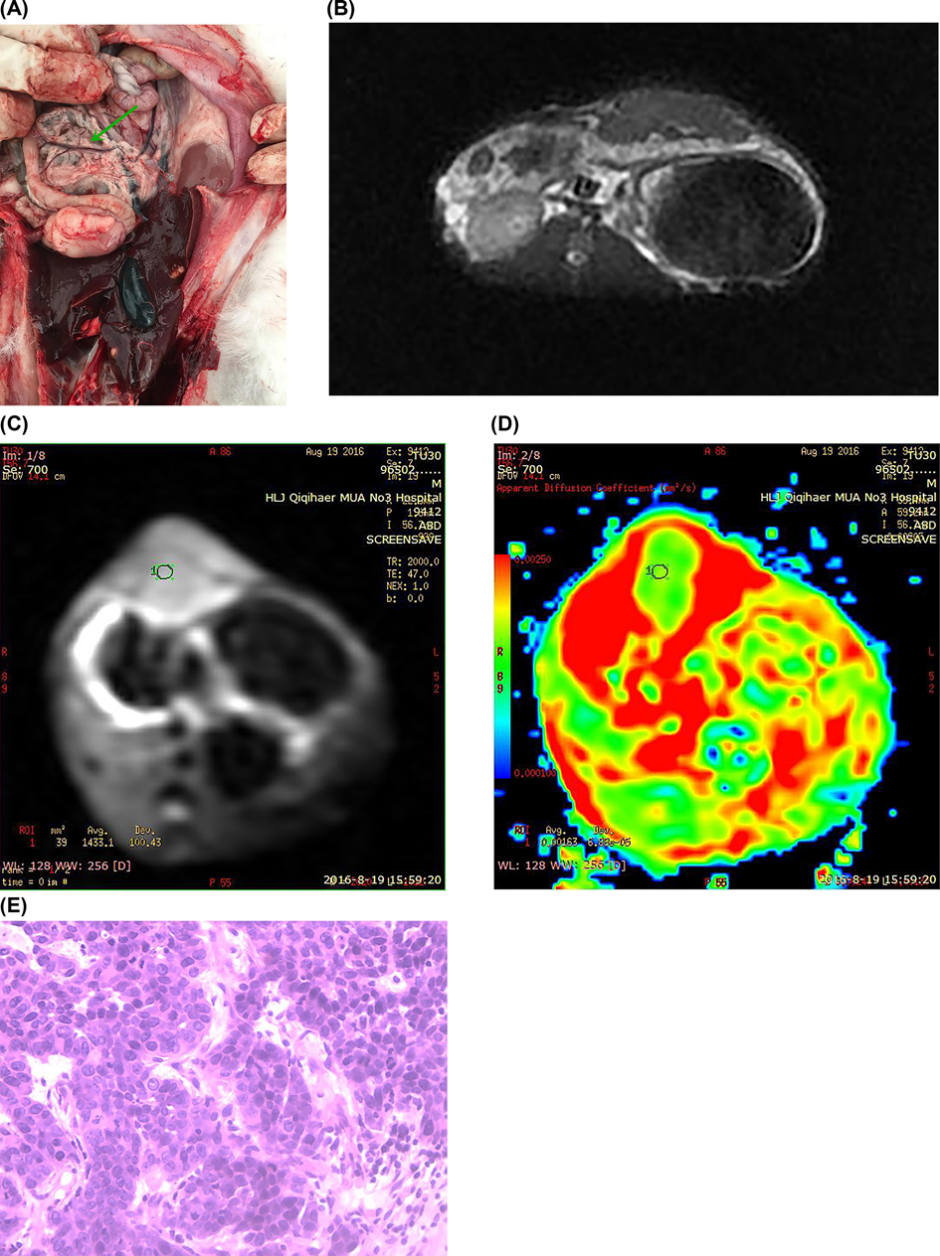
The data of normal pancreatic tissue in control group rabbit (**A**) The yellowish pancreatic tissue was scattered on the lesser curvature. (**B**) T_2_WI image, the pancreas border was clear, and the signal was longer and even. (**C**) DWI image, the signal was uniform and slightly higher. (**D**) b = 333 s/mm^2^, ADC = 1.63. (**E**) Normal pancreatic tissue.

At last, the control group of rabbits were analyzed. The data of normal pancreatic tissue in the control group of rabbits are shown in Figure 4. The yellowish (Figure 4A) pancreatic tissue was scattered on the lesser curvature side of the duodenum, and the pancreatic duct was visible. The fat-suppressing sequence of T2WI is shown in Figure 4B. In this case, the pancreas border was clear, and the signal was longer and even. In the DWI image shown in Figure 4C, the signal was uniform and slightly higher. The b-value was 333 s/mm^2^, and the ADC value (Figure 4D) was 1.63. Figure 3E shows the image of normal pancreatic tissue in a rabbit.

### Repeatability analysis to the duplicate measurement of ADC value

In order to confirm the repeatability of our measurements, three ROIs were selected based on image surface area: ROI_20_ (i.e. a ROI with an area of 20 mm^2^), ROI_46_, and ROI_100_. There was close agreement (ICC > 0.78) in the ADC measurements among ROI_20_, ROI_46_, and ROI_100._ The mean ADCs were not strongly correlated as shown in Table 1.

**Table 1:** Repeatability analysis of duplicate measurement of ADC value

Group	Location	Mean ADC ± SD (×10^−3^ mm^2^/s)	CV	Bias	LOA	ICC
Group 1	ROI_20_	1.82 ± 0.45	10.1%	<0.01	[−0.25, 0.27]	0.87
	ROI_46_	1.80 ± 0.49	8.9%	0.01	[−0.31, 0.24]	0.79
	ROI_100_	1.85 ± 0.47	11.2%	0.03	[−0.26, 0.37]	0.89
Group 2	ROI_20_	2.72 ± 0.37	6.5%	0.01	[−0.19, 0.21]	0.89
	ROI_46_	2.70 ± 0.49	7.8%	0.02	[−0.22, 0.29]	0.83
	ROI_100_	2.68 ± 0.51	6.2%	<0.01	[−0.17, 0.21]	0.84
Control group	ROI_20_	2.35 ± 0.36	6.0%	<0.01	[−0.21, 0.30]	0.79
	ROI_46_	2.32 ± 0.38	6.3%	0.01	[−0.20, 0.23]	0.82
	ROI_100_	2.36 ± 0.37	7.2%	0.02	[−0.16, 0.18]	0.90

### The comparison of MRS metabolism between pancreatic cancer model and pancreatitis model rabbits

Comparison is performed by observing different peaks in the spectra. First, the Lip peak is a lipid peak, caused by cell necrosis in malignant tumors. Furthermore, cell membrane degradation can often be measured with the increase in tumor malignancy, cell necrosis and also caused lipid signal increasing. At the same time, the Lip peak can reflect the intracellular and extracellular fatty acid methylene (–CH_2_–) content.

Second, the Cho peak is the choline complex peak, mainly involved in the composition of cell biofilm and cell biofilm transport. Cho concentration correlates with cell density and cell growth caused by cell division in malignant tumors.

Third, the Cr peak reflects creatine and creatine phosphate levels, and can be used to identify energy metabolism activity. The Cr value does not generally change with pathological changes, so Cr is used in the standardization of the metabolic signal strength. However, it cannot be used as a reference value in the case of highly malignant tumors. When total cholesterol (Chol) levels increase, the content of unsaturated (Unsat) fatty acids and fat content is positively correlated. Therefore, the Chol+Unsat/Lip ratio was used to measure the lipid situation in the rabbit model groups.

In the present study, the appearance of the Lip and Chol+Unsat peaks was relatively stable, and quantitative analysis of peak-to-peak ratio was carried out. However, the appearance of the Cho and Cr peaks in the spectrum were not as stable as they can be influenced by many other factors.

**Figure 5:**
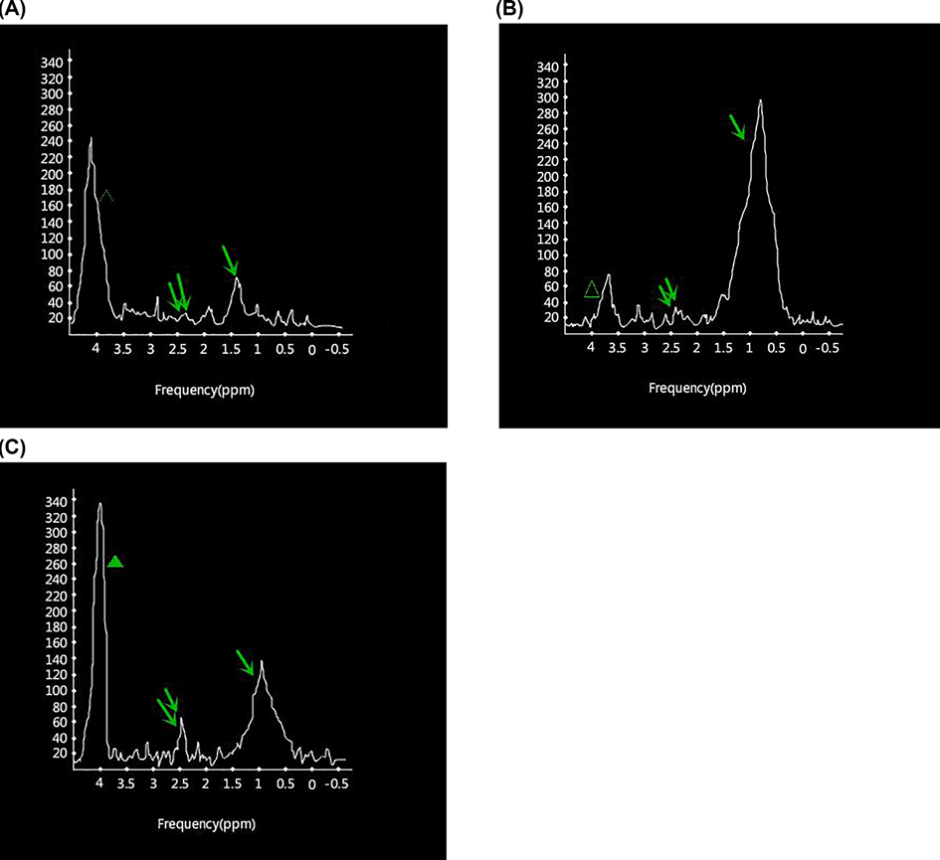
The comparison of MRS metabolism between the three rabbit groups ↑ is Lip peak;↑↑ is Cr peak; Δ is Chol+Unsat peak; ▲ is residual water peak. From left to right: (**A**) 1H-MRS of pancreatic cancer model rabbit. (**B**) 1H-MRS of pancreatitis model rabbit. (**C**) 1H-MRS of normal pancreatic tissue.

In the pancreatic cancer model rabbits (group 1), the Lip peak was mainly located at 0.90–1.80 ppm, and the Cr and Cho peaks from other metabolites were located at 1.80–4.00 ppm. The Lip peak magnitude was low, and the peak of Cho and Chol+Unsat was not significant with a value of Chol+Unsat/Lip = 0.31, as shown in Figure 5A.

In pancreatitis model rabbits (group 2) the Lip peak was 0.90–1.80 ppm, while the Cr and Cho peaks were located at 1.80–4.10 ppm. The Lip peak magnitude in the pancreatitis group was higher than that in the pancreatic cancer group. The peak of Cho and Chol+Unsat was not statistically significant. However, they displayed a difference from the pancreatic cancer models, which had Chol+Unsat/Lip = 0.26, as shown in Figure 5B.

The comparison of ^1^H MRS metabolites display rate is shown in Table 2. The main metabolic peaks in MRS include Lip, Chol+Unsat, Cho and Cr. No statistical significance was found in the comparison of every peak display rate (*P*>0.05).

**Table 2:** The comparison of ^1^H MRS metabolites display rate (%)

Group	*n*	Lip	Chol+Unsat	Cho	Cr
Group 1	8	8 (100.00)	7 (87.5.00)	5 (62.50)	4 (50.00)
Group 2	10	10 (100.00)	9 (90.00)	6 (60.00)	6 (60.00)
Control group	10	10 (100.00)	8 (80.00)	7 (70.00)	7 (70.00)
*F*		-	0.438	0.233	0.749
*P*		-	0.804	0.890	0.688

The comparison between the three rabbit groups for (Chol+Unsat)/Lip indicators is shown in Table 3. Levels of 5.4–5.6 ppm were taken as a baseline to calculate the (Chol+Unsat)/Lip ratio of model rabbits between the three groups. Variance analysis was then performed between the groups (F = −5.736, *P*=0.017), group 1 vs control group (t = −7.027, *P*=0.000), and group 2 vs control group (t = −5.992, *P*=0.015). Overall, statistical significance was found (*P*<0.05).

**Table 3:** The comparison of indicators of Chol+Unsat/Lip among three groups 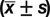

Group	*n*	Chol+Unsat/Lip	95% CI
Group 1	8	0.224 ± 0.095	0.173–0.224
Group 2	10	0.267 ± 0.101	0.196–0.308
Control group	10	0.316 ± 0.125	0.241–0.408
*F*	-	−5.736	-
*P*	-	0.017	-

### The comparison of ADC values among the three groups

The ADC values gradually decreased as b-value increased in the three groups. However, the b-values were quite different from each other. The ADC values in the pancreatic cancer models were significantly lower than chronic inflammation and normal pancreatic areas under the same b-value. Significant difference was also found (*F* = 6.662, *P*=0.014) in the comparison of ADC values within each organ tissue with b-values under 333 s/mm^2^. Table 4 shows the comparison between the groups: both group 1 vs group 2 (*t* = 6.773, *P*=0.003), and group 1 vs control group (*t* = 5.883, *P*=0.016) showed significant difference (*P*<0.05). However, there was no significant difference between groups (*P*>0.05) with b-values under 667 s/mm^2^ and 1000 s/mm^2^.

**Table 4:** The comparison of ADC value between groups (mm^2^/s)

Group	*n*	333 s/mm^2^	667 s/mm^2^	1000 s/mm^2^
Group 1	8	1.83 ± 0.46	1.46 ± 0.22	1.22 ± 0.17
Group 2	10	2.75 ± 0.52	1.94 ± 0.47	1.55 ± 0.23
Control group	10	2.37 ± 0.38	1.86 ± 0.23	1.53 ± 0.22
F		6.662	2.116	0.997
*P*		0.014	0.271	0.872

### The comparison of ADC (DADC) in each group

Table 5 shows the comparison between the groups. As b-value increased, DADC values gradually declined for various tissues. There was statistically significant difference (*P*<0.05) between the pancreatic cancer models and pancreatitis models (*F* = 7.753, *P*=0.000), the pancreatic cancer and normal pancreatic (*F* = 6.634, *P*=0.008) models, and the pancreatitis and normal pancreatic tissue models (*F* = 6.606, *P*=0.010). Bonferroni’s correction was used to correct for the accumulation of the α error, when the comparison between b-value was 333 and 667 mm^2^/s (*P*<0.0167); when the comparison between b-value was 1000 and 667 mm^2^/s, significant difference was not found (*P*>0.0167); when the comparison between b-value was 333 and 1000 mm^2^/s, there was statistically significant difference (*P*<0.0167).

**Table 5:** Comparison of the DADC between each tissue (mm^2^/s)

b-value	Pancreatic cancer and pancreatitis	Pancreatitis and normal pancreatic	Pancreatitis and normal pancreatic
333	1.12 ± 0.19	0.75 ± 0.20	0.39 ± 0.25
667	0.52 ± 0.10	0.36 ± 0.15	0.05 ± 0.02
1000	0.23 ± 0.11	0.15 ± 0.05	0.03 ± 0.01
*F*	7.753	6.634	6.606
*P*	0.000	0.008	0.010
*P_333_* VS*_667_*	0.007	0.012	0.024
*P_667_* VS *_1000_*	0.027	0.036	0.023
*P_333_* VS*_1000_*	0.000	0.000	0.002

## Discussion

At present, domestic and foreign researchers advocate for the regular screening of high-risk groups for pancreatic cancer [10]. They also emphasize the early diagnosis of pancreatic cancer, early radical surgery and early comprehensive treatment. Due to several factors such as the small size of the pancreas, the deep location, the complication of surrounding structures etc, there are few studies on pancreatic animal models, especially in rabbit models. In recent years, with the advancement of high-field magnets [11], high-performance gradient and phased-array surface coils, as well as the improvement of magnetic resonance imaging technology, MRS, DWI, and other new technologies has led to increased study in the diagnosis of pancreatic diseases including pancreatic cancer and pancreatitis. However, the differential diagnosis of pancreatic cancer and pancreatitis is still difficult in clinical work. Based on the rabbit model, a method for diagnosis and differential diagnosis in pancreatic cancer and pancreatitis with MRS and DWI [12] has been successfully proposed in the current study.

Nowadays, there is a consensus in the level of b-value for brain DWI in clinical settings. It is different in the choice for abdominal b-value, and this region does not have a recognized reference b-value [13]. At this stage in the study of pancreatic disease, only some DWI has been performed. However, b-value selection is inconsistent for pancreatic DWI. Since the b-value is a diffusion-sensitive gradient field parameter, it can determine the differences between different tissues in DWI. Theoretically, as b-value increases, the signal decays gradually. In order to reduce the impact of perfusion and other factors on the DWI signal, we selected b-values greater than 150 s/mm^2^ for the study. The larger the b-value, the closer the ADC value is to the true value. The high b-value removes influence of the macroscopic movement of water molecules. The b-value is mainly affected by the organizational structure, tissue composition, cell density within the cell and the integrity of the cell membrane. It can increase the signal to noise ratio (SNR) in a ‘single shot’ EPI by Multi-b-value DWI sequence scan.

In this research, different combinations of b-values were used and diffusion images, ADC, and DADC values of various tissues were compared. MRS was then used to obtain the spectra corresponding to the metabolic status of each tissue. Based on the consideration of tissue metabolism, we explored appropriate b-value DWI settings in pancreas model rabbits [14].

The tumor microenvironment in rabbits is similar to humans and thus it can be effective to have the simulation studies in the local lesion and distant metastasis of pancreatic cancer. In the present study, good results were obtained in the orthotopic transplantation of VX-2 tumor, the modeling success rate was 100%. However, this method also has many drawbacks such as complex operation, high costs, large damage to the pancreas, postoperative recovery time, and high mortality (20% in the present study). Successfully obtaining a good model of mass pancreatitis by ligation of the main pancreatic duct is also achieved. A normal primary pancreatic duct can be seen clearly as shown in Figure 4A, and the success rate is 100%. The present study provides a modeling method for the establishment of pancreatic cancer and lump pancreatitis in rabbits, and provides reference for researchers engaged in basic study of pancreas.

No significant difference was found in plain scans, i.e. T_1_WI and T_2_WI between pancreatic cancer and mass pancreatitis. This scan can be used as a basis for other scanning sequences. In T_1_WI images, normal pancreatic tissue showed a higher signal due to the abundance of watery proteins. Mass pancreatitis showed slightly lower or equal signal, and at last, pancreatic cancer showed low or slightly low signal. In the T_2_WI images, mass pancreatitis showed equal or slightly higher signal, with pancreatic cancer showing equal or higher signal. However, high signal was observed in serious necrosis. Fat-suppressed imaging is proposed as a method to improve the sensitivity and specificity of identifying pancreatic sites (as shown in Figures 2B, 3B, and 4B for pancreatic cancer, mass pancreatitis, and normal tissues, respectively). However, the tumor often causes changes in the parenchyma around the lesion. Therefore, fat-suppressed imaging is not recommended for qualitative differential diagnosis.

DWI, as a functional imaging option, obtains the image by detecting the body’s irregular (random) movement of water molecules (as shown in Figures 2C, 3C, and 4C for pancreatic cancer, mass pancreatitis, and normal tissues, respectively). In the present study, DWI imaging was performed on rabbit pancreas occupancy models. DWI does not only show intracellular water molecules and water molecule transmembrane movement, but also the tissue perfusion and extracellular water molecular movement. Therefore, as the b-value increases, the ADC value will be changed in the pancreatic cancer area, the inflammatory mass area, and the normal pancreatic tissue.

Pancreatic cancer cells are extracted from the ductal epithelium. Therefore, pseudopapillary structure may be observed. The cancer cells are differentiated as wedge-shaped or polygonal, as having eosinophilic granules in the cytoplasm, and from the formation of acini or small clumps. Pathological changes in pancreatitis includes pancreatic interstitial fibrosis or extensive fibrosis, acinar and pancreatic tissue atrophy, chronic inflammatory cell infiltration, and mucus plug formation and calcification in the pancreatic duct. It is a technically challenge to distinguish pancreatitis from pancreatic cancer in general pathology due to its hard texture and fibrosis formation.

^1^H-MRS can detect and quantitatively analyze Lip, Cho, Chol+Unsat and Cr metabolites in the normal pancreas of the living body. Metabolic peaks appear in varying degrees in the spectra of pancreatitis and pancreatic cancer. The peak display rate results in Table 1 show the appearance of the Lip and Chol+Unsat peaks as relatively stable. In the present study, relative quantitative analysis of the peak height ratio shows that the appearance of Cho and Cr peaks in the spectrum is not stable. It can be affected by many other factors. Therefore, the author compared the display rate between groups of metabolites.

There is diagnostic value of Cho peak changes in malignant lesions, because increasing Cho content reflects the increase in cell membrane composition and the acceleration of cell proliferation. Lip peak level reflects the intracellular and extracellular fatty acid methylene (–CH_2_–) content. The lipid content is abundant in pancreatic gland cells and interstitial cells, and there is a high lipid (Lip) peak in the normal pancreatic tissue spectrum. Considering that pancreatitis and pancreatic cancer mass lesions can replace the normal pancreatic tissue, while reducing the normal pancreas fat composition, this may be the reason for the decreased levels of Lip in chronic pancreatitis and pancreatic cancer.

The reason for low peak of lip in pancreatic cancer can be attributed to several factors. First, the main components of pancreatic cancer are the different density of tumor cells and the large number of fibrous matrices. Therefore, Lip content was found to be significantly lower than the normal lipid-rich glands. Second, tumor tissue cells increase water content due to the need for increased metabolic levels. Third, high tumor metabolism and high energy consumption environments are not conducive to the accumulation of lipid energy substances. At last, water precession frequency changes in tumor cells due to water and biological macromolecule interactions, e.g. non-uniform tumor tissue and internal magnetic field interaction. This reduces the efficiency of the selective saturation pulse on the suppression of water signals in the tumor [15].

Some studies show that cholesterol esters associated Chol may increase in certain malignancies [16]. This mechanism needs to be further explored. Unsaturated content and fat content are positively correlated. Therefore, the change of unsaturated fatty acid to fat does not affect the result of Chol+Unsat/Lip ratio. In the present study, the ratio of Chol+Unsat/Lip in chronic pancreatitis and pancreatic cancer group gradually increased as shown in Table 3. This may be associated with the gradual reduction in fat in pancreatic lesions. There is no statistically significant difference between the pancreatitis and pancreatic cancer models, revealing some essential connection between the two diseases. However, further research is required, taking into account the increase in the Chol+Unsat/Lip ratio as the characteristic marker in diagnostic pancreatic cancer.

Overall, ADC values gradually decreased as b-value increased in all the three groups, even though the b-value was different in each group. The ADC value of pancreatic cancer was significantly lower than chronic inflammation and normal pancreatic tissue under the same b-value. There was a significant difference (*F* = 6.662, *P*=0.014) in the comparison of ADC values within each organization under 333 s/mm^2^ b-value (Table 4). A comparison was undertaken between the groups: group 1 vs group 2 (*t* = 6.773, *P*=0.003), group 1 vs control group (*t* = 5.883, *P*=0.016), also had significantly difference(*P*<0.05). There was no significant difference between the groups (*P*>0.05) with b-values under 667 s/mm^2^ and 1000 s/mm^2^. It was also noted that the ADC value for pancreatitis and pancreatic cancer gradually decreased with the increase in b-value. This is mainly due to tissue changes in fibrosis, resulting in reduced pancreatic blood flow and water, and thus decreased perfusion and low b-value (333 s/mm^2^), which is more sensitive to perfusion in this research.

In different tissues, and pathophysiological processes of the same tissue, the ADC value is not the same. In addition, the DADC value was different in different stages of the same lesion and different lesions of the same organ tissue. It is closely related to the perfusion of the organization. As shown in Table 4, whereby as b-value increased, DADC values gradually declined among various organizations. As b-value increased, there was statistically significant difference (*P*<0.05) in pancreatic cancer and pancreatitis (*F* = 7.753, *P*=0.000), pancreatic cancer and normal pancreatic tissue (*F* = 6.634, *P*=0.008), pancreatitis and normal pancreatic tissue (*F* = 6.606, *P*=0.010). For b-values of 333 and 1000 mm^2^/s, there was a statistically significant difference (*P*<0.05) in the comparison between groups: pancreatic cancer and pancreatitis (*t* = 6.703, *P*=0.007), pancreatic cancer and normal pancreatic tissue (*t* = 5.594, *P*=0.015), pancreatitis and normal pancreatic tissue (*t* = 5.018, *P*=0.021). Therefore, by comparing with higher b-values (1000 mm^2^/s), the difference in DADC can be obtained by which the ADC value at lower b-values (333 s/mm^2^) is detected.

In summary, with the application of this methodology, it is feasible to differentiate pancreatic cancer from pancreatitis by applying different high b-values and low b-values. The present study measured the ADC value for pancreatic cancer as lower compared with normal pancreatic tissue at different b-values. There is no significant difference in the comparison of DADC, since with the decrease in b-value, the DADC value of both increases. Thus, there are some differences in molecular diffusion and tissue perfusion between pancreatic cancer and pancreatitis under MR molecular imaging in combination with the DWI techniques as described in the present study.

## Conclusion

The appearance of the Lip and Chol+Unsat peaks was relatively stable as determined from ^1^H-MRS images, but these cannot necessarily be used to identify pancreatic cancer and pancreatitis model rabbits. Although larger b-values, correspond to closer real tissue ADC values, low b-values (333 s/mm^2^) should be selected to obtain DADC, and therefore obtain a good value in differentiating the diagnosis of pancreatic cancer and pancreatitis in rabbit models.

## Abbreviations

ADC: Apparent Diffusion Coefficient
Chol: Total Cholesterol
Cr: Creatine
DADC: Difference of ADC
DWI: Diffusion-weighted Imaging
EPI: Echo Planar Imaging
FOV: Field of Vision
HE: Hematoxylin-eosin staining
ICC: Interclass Correlation Coefficient
Lip: Lipid
MRS: Magnetic Resonance Spectroscopy
ROI: Region of Interest
T_1_WI: T_1_ Weighted Imaging
T_2_WI: T_2_ Weighted Imaging
Unsat: Unsaturated
VOI: Volume of Interest
